# Behavioral Determinants of Childhood Obesity in the United States: An Exploratory Study

**DOI:** 10.1155/2024/9224425

**Published:** 2024-11-15

**Authors:** Soumitra Palit, Tahia Sufyani, Joseph N. Inungu, Chin-I. Cheng, Emmanuel Nartey

**Affiliations:** ^1^College of Public Health-Epidemiology Concentration, University of New Mexico, Albuquerque, New Mexico, USA; ^2^Department of Public Health, Central Michigan University, Mount Pleasant, Michigan, USA; ^3^Department of Medicine and Surgery, Sylhet M.A.G Osmani Medical College, Sylhet, Bangladesh; ^4^Department of Statistics, Actuarial and Data Science, Central Michigan University, Mount Pleasant, Michigan, USA

## Abstract

Childhood obesity is a complex and multifactorial phenomenon. Understanding these factors is crucial in developing effective interventions to prevent and treat childhood obesity. The purpose of this study is to provide an update on factors related to childhood obesity in the United States. This cross-sectional study analyzed data from the 2021 Youth Risk Behavior Surveillance System (YRBSS) survey to assess factors associated with childhood obesity among US children aged 12 to 17 years. Logistic regression analysis was used to identify the sociodemographic factors associated with overweight and obesity. Data were analyzed using *R* studio (4.3.2). A total of 12,836 respondents were enrolled in this study. Among them, the prevalence of overweight, obesity, and morbid obesity was found to be 17.66%, 11.21%, and 1.76%, respectively. Respondents with a BMI over 25 were mostly male (17.63%) and of White race (32.77%). The main sociodemographic factors associated with overweight and obesity were being 14 or 15 years old, male, non-White, having a history of alcohol or marijuana consumption, and not practicing physical activity. These findings can inform targeted interventions for prevention and management. This research sheds light on critical sociodemographic factors related to childhood obesity in the U.S., highlighting its complexity. The findings emphasize the influence of age, gender, ethnicity, and lifestyle behaviors, such as substance use and physical inactivity, on obesity rates among youth. These insights are crucial for developing targeted interventions. Addressing these factors offers a real chance to enhance future health outcomes, and underscoring the need for comprehensive strategies that include both health education and broader community support to instill healthy habits early on. In addition, unexpected results concerning vegetable consumption and the omission of genetic and familial data suggest areas for further research.

## 1. Introduction

Childhood obesity is a serious public health problem in the United States, putting children and adolescents at risk for poor health [1]. According to the CDC, the prevalence of obesity among children and adolescents was 19.7% in 2022, affecting an estimated 14.7 million children. Childhood obesity is more common in certain populations. About 26.2% of Hispanic children, 24.8% of non-Hispanic Black children, 16.6% of non-Hispanic White children, and 9.0% of non-Hispanic Asian children suffered from childhood obesity. Childhood obesity significantly impacts both their physical and psychological health. Obesity can lead to severe health conditions, including non–insulin-dependent diabetes mellitus (NIDDM), cardiovascular problems, bronchial asthma, obstructive sleep apnea (OSA), hypertension (HTN), hepatic steatosis (fatty liver disease, FLD) gastroesophageal reflux (GER), and psychosocial issues [2].

Several studies [3, 4] suggest that environmental factors such as lack of access to healthy food options and safe spaces for physical activity play a critical role in the development of childhood obesity. Furthermore, the consumption of high-calorie diets and reduced physical activity levels have also been linked to the development of childhood obesity. Studies suggest that variations in certain genes can make individuals more susceptible to obesity. However, these genetic factors are not entirely deterministic [5].

A previous study by Jia. P et al. [6] reported that fast food consumption as well as its availability is associated with a higher risk of obesity in the community. Media exposure or prolonged screen time also plays a vital role in childhood obesity. Stanford et al. [7] reported that media exposure is positively associated with obesity. On the other hand, Wen et al. [8] found that screen time and sleep duration were contributing factors to childhood obesity. Sleep time duration is considered an important factor for childhood obesity. Sánchez-López et al. [9] found that children with later bedtimes or irregular sleep schedules may be more likely to develop obesity. Children who had gone through adverse childhood experiences tend to get more depressed, which can lead to obesity. Kyler et al. [10] reported a strong association between exposure to adverse childhood experiences (ACEs) and obesity risk in childhood. Mental stress and anxiety are associated with childhood obesity. Miller et al. [11] reviewed 24 studies on stress-related childhood obesity and reported that stress in early childhood was associated with an increased risk of obesity, which is also a positive finding in our study.

Given the significant public health consequences of childhood obesity, it is essential to identify the factors that contribute to its development. Identifying these factors can assist in the development of targeted interventions and policies to prevent or reduce the prevalence of childhood obesity in the United States and improve children's overall health and well-being.

### 1.1. Conceptual Framework

**Figure 1:**
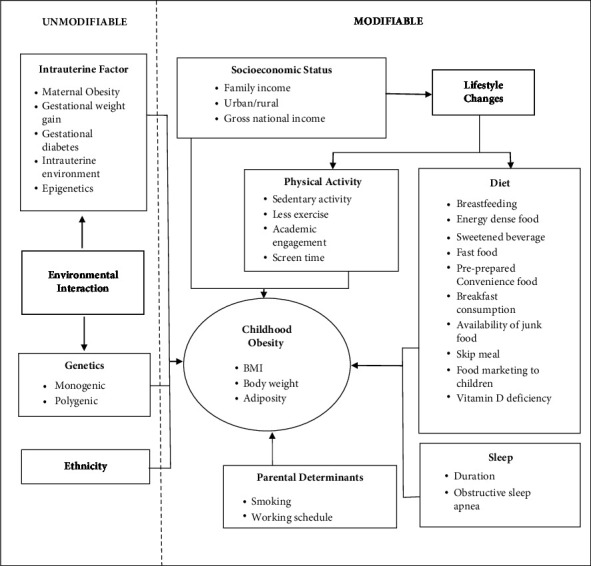
Multifactorial influences of childhood obesity. Source: Ang et al. [12]. Reprinted from Multifactorial Influences of Childhood Obesity, authored by Ang, Y. N., Wee, B. S., Poh, B. K., & Ismail, M. N. (2012). Permission to use this figure was granted by the publisher under a licensing agreement (license number: CC-BY-NC-SA 5737700641356).

The conceptual framework by Ang et al. [12] guided this review (see Figure 1). Variables described in this conceptual framework were included in our study. Another cross-sectional study was carried out by Wethington et al. [13]; their research explored factors such as physical activity, consumption of energy-dense foods, intake of fruits and vegetables, and the consumption of sugar-sweetened beverages. These prior studies serve as a foundation for identifying and examining relevant variables in this research.

The framework presents factors that are unmodifiable—such as genetics, ethnic differences, gestational weight, and intrauterine conditions, as well as modifiable factors, such as socioeconomic status, diet, physical activity, sleep, and parental determinants.

Ang et al.'s [12] paper titled “Multifactorial Influences of Childhood Obesity” explores the diverse factors contributing to childhood obesity. They delve into the genetic aspect, highlighting rare but severe conditions associated with single-gene mutations. The paper emphasizes that obesity can also result from the interaction of multiple genes regulating various aspects such as hunger, satiety signals, adipocyte growth, and energy expenditure. Ethnic differences are discussed, with a focus on body composition and fat distribution variations among different groups. The authors stress the impact of cultural beliefs, perceptions, and activities on childhood obesity disparities. The gestational and intrauterine factors' section underscores the significance of maternal obesity during pregnancy, linking it to an increased risk of childhood obesity. The authors elaborate on the role of gestational weight gain and in utero exposure to excess energy, hormones, and growth factors, emphasizing their influence on epigenetic modifications. Socioeconomic status (SES) is identified as a contributing factor, with higher SES associated with an increased risk due to greater access to energy-dense diets. The paper acknowledges the variation in the association between obesity and SES based on gender, age, and country. Dietary habits are explored, with breastfeeding highlighted for its protective effect against childhood obesity. The adoption of Western lifestyles is discussed concerning increased intake of energy-dense foods. The importance of regular and balanced meals, especially breakfast, is emphasized as a preventive measure. Physical activity is underscored as essential for preventing childhood obesity, while sedentary behaviors, including excessive screen time, are identified as contributors to increased risk. The paper addresses sleep duration and its association with increased obesity risk, attributing it to hormonal alterations and behavioral changes. The predictive role of visceral adiposity in OSA among obese children is highlighted. Lastly, the authors discuss parental determinants, including maternal smoking during pregnancy, exposure to nicotine, higher parental BMI, and nonstandard work schedules, emphasizing their impact on children's diet and physical activity. Overall, Ang et al. [12] provide a comprehensive overview of the multifactorial influences on childhood obesity, encompassing genetic, ethnic, gestational, socioeconomic, dietary, physical activity, sleep-related, and parental factors in their analysis.

The abovementioned summary of the article guided this research to determine the variables that are feasible for the secondary data source and research question.

## 2. Methodology

### 2.1. Study Design and Sampling

We analyzed data from the 2021 Youth Risk Behavior Surveillance System (YRBSS), a cross-sectional survey study of youth in the USA. The YRBSS-2021 collects data on a wide range of information to monitor childhood behaviors including health behaviors, sexual behavior, physical activity, and nutrition. YRBSS is an important tool for researchers, policymakers, and public health professionals who are interested in promoting the health and well-being of youth in the United States. Although the YRBSS does not track the health outcome of behavior in the same participants over time, it collects data from a representative sample of the population of interest to estimate the outcome or behavior in the overall population.

### 2.2. Sample Size

A total of 12836 participants were recruited. A pretested questionnaire was used to collect the following information.

### 2.3. Demographics and Contextual Variables

Three major types of information were collected: demographic, behavioral, and clinical variables.

Demographic data included participant's age (12–17 years), sex (male and female), race (Black African, Asian, American Indian, or Alaskan Native, Native Hawaiian or another Pacific Islander, White, and multiple races).

### 2.4. Youth Risk Behaviors for Obesity

The YRBSS collected information on participants' smoking habits, alcohol consumption, marijuana consumption, vegetable and fruit consumption, habit of weekly soft drink consumption, involvement in physical activity, screen time, sleeping time, ever experienced bullying, attitude towards change in weight status, and sexual behavior. Finally, clinical information was collected based on the participant's depressive mood, regularity of oral health checkups, mental health issues, and BMI status: All the participants' height and weight were measured, and the BMI category was calculated based on its level. The normal BMI range is 18.5–24.9 kg/m^2^. Those who are <18.5 kg/m^2^ are regarded as underweight, those within the range of 18.5–24.9 kg/m^2^ are regarded as normal body weight, those within the range of 25-29.9 kg/m^2^ are regarded as overweight, and those who are >30 kg/m^2^ are regarded as obese.

### 2.5. Statistical Analysis

Descriptive and bivariate analyses were conducted to describe the characteristics of the participants and the prevalence of obesity and related risk behaviors. A logistic regression test was done to determine the predicted BMI factor. The presence of obesity was the outcome of interest.

### 2.6. Ethical Consideration

As we used the YRBSS as our secondary data source, ethical considerations for the YRBSS include the following:

Privacy and confidentiality: using anonymous surveys and removing personally identifiable information

Informed consent: obtaining permission from schools and parents and informing students about the survey's purpose and voluntary nature

Responsible data use: considering the potential impact of data on participants and the wider population, using it to address disparities without stigmatization or harm

## 3. Result

**Figure 2:**
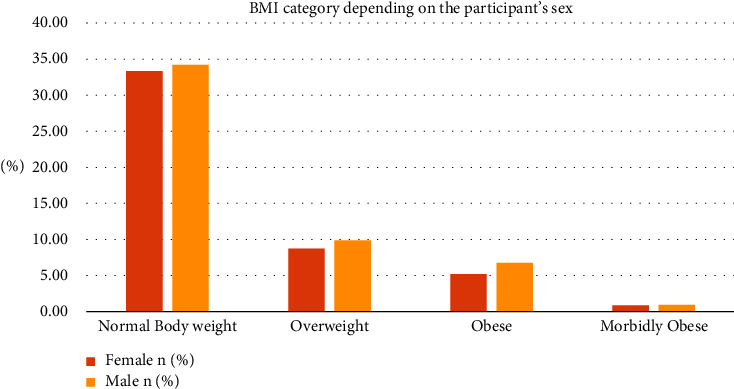
Graphical representation of the BMI category depending on the participant's sex.

The sociodemographic characteristics of the 12,836 participants are shown in Table 1. Among these participants, 48.14% were female. About 25.8% of males and 27.4% of females were 15-16 years old. Among 12,836 participants, 33.33% of females and 34.21% of males fall into the normal body weight category, around 8.72% of females and 9.90% of males are in the overweight category, 5.20% of the females and 6.78% of males are obese, and both males and females have approximately 1% representation in the morbidly obese category (see Figure 2).

**Figure 3:**
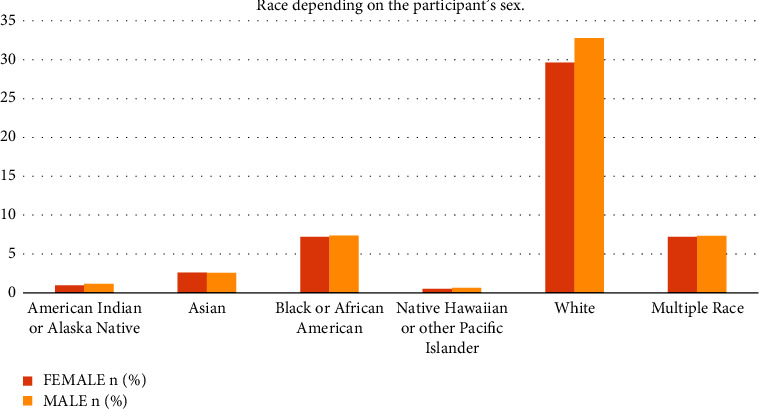
Graphical representation of the race depending on the participant's sex.

With regards to race, the majority of female participants were White, accounting for 29.63% of the total, followed by African Americans at 7.22% and multiple races at 7.20%. Asian, Native Hawaiian or other Pacific Islander, American Indian, or Alaskan Native constituted 2.60%, 0.51%, and 0.96% respectively (see Figure 3). Among male respondents, 32.77% were White and 7.37% were Black African American respondents. The Native Hawaiian or other Pacific Islander group and American Indian and the Alaskan native group showed a result of 0.63% and 1.14%, respectively (see Figure 3).

Considering dietary habits, a notable majority in both genders engage in fruit consumption (41.27% for females and 43.45% for males) and vegetable consumption (27.84% for females and 28.73% for males).

In terms of sexual behavior, a substantial portion of the population is not sexually active, with 23.75% of females and 24.11% of males falling into this category. Active individuals account for 11.59% of females and 11.43% of males.

The results of the bivariate analyses or unadjusted regression analyses for BMI status among children (12–17 years of age) in *R* studio are shown in Table 2. All the variables studied were significantly associated with overweight/obesity. For example, adverse childhood experiences, depression, cigarette smoking, screening, and sleeping time were associated with overweight/obesity.

Table 3 presents the result of the stepwise multivariable logistic regression analysis. The associations between independent variables and the outcome (BMI) were assessed, as indicated by their respective *p* values. Notably, children aged 14 years were significantly less likely than those who were 17 years old to be obese (OR = 0.560; 95% confidence interval (CI): 0.456–0.6860). Children aged 15 years were significantly less likely than those who were 17 years old to be obese (OR = 0.788; 95% CI: 0.660–0.939). Children who were male significantly more likely than those who were female to be obese (OR = 2.352; 95% CI: 2.035–2.718). Children who were American Indian or Alaskan Native (OR = 1.628; 95% CI: 1.075–2.464), Black or African American (OR = 1.980; 95% CI: 1.648–2.379), multiple race (OR = 1.203; 95% CI: 1.001–1.445), and Native Hawaiian or other Pacific Islander (OR = 1.963; 95% CI: 1.073–3.591) significantly more likely than those who were White to be obese. However, Asian students were less likely to be obese than White students (OR = 0.406; 95% CI: 0.295–0.559). Children who consumed alcohol were less likely to be obese than those who did not consume alcohol (OR = 0.676; 95% CI: 0.537–0.850). Children who consumed marijuana were significantly more likely to be obese than those who did not consume marijuana (OR = 1.229; 95% CI: 1.017–1.485). Children who wanted to gain weight (OR = 0.041; 95% CI: 0.031–0.054) were significantly less likely to be obese than those who wanted to lose weight.

Children who were regularly physically active (OR = 0.634; 95% CI: 0.524–0.767) and those who were moderately physically active (OR = 0.737; 95% CI: 0.603–0.900) were significantly less likely to be obese than those who were not physically active. Children who were regular about their oral health checkups (OR = 0.687; 95% CI: 0.547–0.864) and those who were occasional about their oral health checkups (OR = 0.883; 95% CI: 0.631–1.237) were significantly less likely to be obese than those who were not regular about their oral health checkups.

## 4. Discussion

This exploratory study was conducted to provide an update on the behavioral factors associated with childhood obesity in the USA. The results of this study showed that several sociodemographic and behavioral factors were associated with the prevalence of obesity among the study participants. Specifically, adverse childhood experiences (bullying), depression, smoking, use of marijuana, soft drinks consumption, physical activity, screen time, oral health, mental health, and sleeping time were associated with obesity.

Blasingame et al. [14] analyzed a larger dataset from the 2016–2020 National Survey of Children's Health, emphasizing the cumulative impact of multiple social determinants of health (SDOH) domains, finding that high-risk SDOH profiles significantly increase obesity risk. In contrast, this study focused on specific sociodemographic factors, identifying that being 14 or 15 years old, male, non-White, having a history of substance use, and lack of physical activity were major contributors to overweight and obesity among 12,836 respondents aged 12–17 years. Both studies found similar prevalence rates of overweight and obesity and called for multilevel intervention strategies, with Blasingame et al. advocating for comprehensive approaches addressing health equity across multiple SDOH domains, and this study recommending targeted behavioral change strategies at individual, community, and policy levels.

The authors in [15] in 2018 said that stress, anxiety, and depression may lead to calorie-dense junk food consumption to alleviate uncomfortable psychological and emotional states, which play a critical role in weight gain and obesity. Our study also found that depression (*P* < 0.01), anxiety, and stress (*P* < 0.01) were significantly associated with childhood obesity.

Ortega et al. [16] reported that the lack of physical activity was associated with obesity. It is associated with increased abdominal adiposity. This finding is in line with our finding which shows a significant association between lack of physical activity and obesity (*P* < 0.01).

Unexpectedly, our study revealed that children who consumed vegetables had a higher chance of being obese than those who did not. It is probable that children who ate a lot of vegetables also consumed higher-calorie foods such as soft drinks and were less physically active. There is another study carried out by Ledoux et al. [17], which found consuming more fruits and vegetables (FV) together with other behaviors appears to be associated with decreased obesity in adults. On the other hand, children do not exhibit this relationship. In 2018, the authors in [18], in their systematic review, found moderate quality evidence for an inverse association between vegetable intake and weight-related outcomes in adults. It is still unclear if increased FV intake on its own, in the absence of calorie intake or physical exercise, causes decreases in adiposity or slower growth. In our study, we also wanted to determine the association between vegetable consumption and obesity, but it might also have to do with the fruits and vegetables type, quantity, and calories, as well as the physical activity of children and their genetics who consume vegetables, which are not given any thought. The *p* value associated with the factor association would have been different if those behavioral factors had been taken into account.

Childhood obesity is influenced by a complex interplay of factors, including genetics, parental influences, and lifestyle choices. Genetics is an important factor that can influence body weight significantly. A recent study carried out by Wen Dai et al. [19] found that reducing calcium/calmodulin-dependent protein kinase-2 (CAMK2) activity in fat cells might help improve blood sugar control in obesity without needing to lose weight. This study suggests that targeting CAMK2 could be a new way to treat metabolic problems associated with obesity. Within the suggested Ang et al. [12] framework, contextual factors such as cultural norms, community environments, and healthcare access play pivotal roles. Cultural norms shape dietary preferences and body image perceptions, while community environments impact physical activity options and food availability. Access to healthcare influences preventive measures and management strategies. Understanding how these contextual factors intersect with established variables is crucial for designing effective interventions. It allows for tailored approaches that consider cultural diversity, community dynamics, and healthcare accessibility in combating childhood obesity.

In the pursuit of combating childhood obesity, a series of evidence-based interventions unfolded, each building upon the last. In 2007, the authors in [20] laid the groundwork with a focus on community-based interventions. Their study illuminated the effectiveness of collaborative efforts involving diverse stakeholders such as communities, healthcare providers, and policymakers. This underscored the need for a comprehensive strategy, recognizing that lasting changes in environments require multifaceted involvement. Expanding on this community-centric approach in the same year, the authors in [21] shifted the spotlight to school-based interventions. Their work showcased the effectiveness of nutrition education and physical activity programs, positioning schools as pivotal environments shaping children's behaviors and paving the way for healthier lifestyles. In 2011, the authors in [22] broadened the perspective by emphasizing the role of policy interventions. Their systematic review highlighted the impact of measures such as sugar-sweetened beverage taxes and front-of-package labeling in reducing childhood obesity rates. This emphasized the potency of policy advocacy in reshaping obesogenic environments and fostering population-wide change. Moving to 2015, the authors in [23] contributed a practical school-based intervention, encouraging water consumption during school lunch. This not only demonstrated the feasibility of implementing changes within school settings but also addressed the critical aspect of beverage choices. In 2016, the authors in [24] delved into community-level factors, offering insights into influences on dietary habits in low-income urban youth. This knowledge contributed to the development of community-based interventions tailored to address specific contextual factors. Considering socioeconomic factors in the same year, the authors in [25] assessed the impact of a sugar tax on beverages, providing a nuanced perspective. The study underscored the importance of tailoring policy design to consider economic disparities, ensuring interventions are equitable and inclusive. Advocating for family-based interventions in 2017, the authors in [26] emphasized positive outcomes when involving the child in the process. This approach recognized the family unit as a fundamental influencer of a child's behaviors, acknowledging the need for interventions addressing the dynamics within households. In 2020, the authors in [27] recognized the digital era, exploring the potential of technology-driven interventions. Their examination of mobile health applications for pediatric obesity signaled the need for innovative approaches leveraging technology to engage and educate children in the battle against obesity. Further highlighting the impact of policy in the same year, the authors in [28] evaluated Chile's food labeling and advertising law, underscoring the effectiveness of regulatory changes and reinforcing the importance of targeted policy measures in shaping dietary patterns among children. The most recent addition to this evolving narrative in 2023 was the study by authors in [29], who delved into family-based interventions within routine pediatric care. Their study not only reaffirmed the effectiveness of such interventions but also provided valuable insights into different treatment groups, paving the way for nuanced and personalized approaches. This chronological sequence paints a comprehensive picture of interconnected interventions over the years in the fight against childhood obesity.

Keeping back all these findings in mind, we suggest some potential avenues for future research related to childhood obesity. Firstly, investigating the effectiveness of digital health interventions, such as mobile apps and wearable devices, in promoting physical activity and healthy eating habits among children offers promise. Secondly, understanding the impact of interventions during prenatal and early childhood periods on long-term obesity outcomes is crucial for developing targeted prevention strategies. Thirdly, delving into the role of genetic and epigenetic factors in childhood obesity, with a focus on personalized interventions, is an emerging area. In addition, examining the influence of community engagement and the built environment on children's habits can inform the development of effective community-based interventions. Lastly, targeted interventions should be developed to address the varying behavioral risk factors among different sociodemographic groups. For instance, programs that focus on reducing obesity among older adolescents and males may be particularly beneficial along with considering the influence of dietary preferences, substance use, and physical activity on obesity, health education, and interventions should promote both healthy behaviors and weight perception. Conducting long-term follow-up studies on existing childhood obesity interventions is imperative to assess sustained impacts into adolescence and adulthood.

## 5. Limitations

While the YRBSS provides valuable insights into the health behaviors of high school students, there are some limitations to consider when interpreting and using these data. Some of the limitations include the following.

Self-reporting bias: Respondents may underreport or overreport certain behaviors due to social desirability bias or recall bias. This can lead to an underestimation or overestimation of the prevalence of certain health behaviors.

Sampling bias: The YRBSS collects data from a sample of high-school students, which may not fully represent the entire population of youth in the United States. It is important to consider the demographic characteristics of the sample and recognize that the findings may not be generalizable to all high-school students or youth populations.

Nonresponse bias: The characteristics of nonrespondents may differ from those who participate, potentially affecting the representativeness of the data.

Cross-sectional design: The YRBSS is a cross-sectional study, collecting data at a single point in time. This limits the ability to establish causal relationships or examine changes in behavior over time.

Limited scope: The YRBSS focuses on specific health behaviors and risk factors, but it does not capture the full spectrum of factors that influence youth health and well-being. Other important aspects, such as mental health, socioeconomic factors, and environmental influences, are not comprehensively assessed by the YRBSS.

Reliance on self-perception: The YRBSS relies on students' self-perception of their health behaviors, which may not always align with objective measures. For example, students may overestimate their physical activity levels or underestimate their weight status.

## 6. Novelty and Contribution of the Study

The utilization of a substantial and representative sample of individuals in the United States (*N* = 12,836) constitutes a major strength of this research endeavor. This investigation delved into the various factors influencing childhood obesity by incorporating a broad spectrum of behavioral and sociodemographic elements, thereby shedding light on novel findings that challenge prevailing perspectives. Noteworthy is the inclusion of oral health in the analytical framework as a pivotal variable, presenting a new angle on its implications for body weight. Furthermore, an in-depth examination of psychological dimensions was undertaken, encompassing an evaluation of the impact of adverse childhood experiences, depression, and behavioral intentions concerning weight management, thereby unveiling fresh insights into the mental health aspects of obesity. The meticulous scrutiny of sociodemographic determinants, such as age, gender, and ethnicity, contributes significantly to the comprehension of the multifaceted character of obesity. Through the consideration of the combined impacts of these diverse elements, the study propounds nuanced suggestions for tailored and personalized intervention approaches, signifying a notable progression in the realm of childhood obesity scholarship. Nonetheless, in contrast to prior investigations, this study brought to light a paradoxical association between heightened vegetable consumption and an elevated body mass index (BMI). The researchers encountered difficulties in providing a definitive rationale for this discovery, which is acknowledged as a constraint of the present study.

## 7. Conclusion

This research has brought to light important sociodemographic factors associated with childhood obesity in the United States, emphasizing the complex nature of this public health issue. The results draw attention to the critical roles of age, gender, ethnicity, and lifestyle habits, including substance abuse and lack of physical activity, in the levels of overweight and obesity among young individuals. These observations are crucial for policymakers, healthcare professionals, and community stakeholders in designing evidence-based interventions aimed at addressing the increasing prevalence of obesity among children. By addressing these factors, we have a real chance of improving the health of future generations. This emphasizes the need for integrated strategies that encompass not only health education but also support from our environment and community to encourage healthy habits from a young age. However, the counterintuitive findings related to vegetable consumption and the lack of information on genetic factors and family history call for more in-depth research to better understand the mechanisms at play.

## Figures and Tables

**Table 1:** BMI distribution by sex (total sample number 12836).

Variables	Female *n* (%)	Male *n* (%)
*BMI category*		
Normal	4278 (33.33%)	4392 (34.21%)
Overweight	1120 (8.72%)	1271 (9.9%)
Obese	667 (5.20%)	871 (6.78%)
Morbidly obese	115 (0.89%)	122 (0.95%)

*Age*		
≤12	5 (0.04%)	4 (0.03%)
13	20 (0.15%)	17 (0.13%)
14	1220 (9.50%)	1282 (9.99%)
15	1692 (13.18%)	1771 (13.79%)
16	1620 (12.62%)	1884 (14.67%)
17	1623 (12.64%)	1698 (13.23%)

*Race*		
American Indian or Alaska Native	123 (0.96%)	147 (1.14%)
Asian	335 (2.60%)	332 (2.58%)
Black or African American	927 (7.22%)	946 (7.37%)
Native Hawaiian or other Pacific Islander	66 (0.51%)	81 (0.63%)
White	3804 (29.63%)	4207 (32.77%)
Multiple race	925 (7.20%)	943 (7.34%)

*Dietary habit*		
Fruit consumption: yes	5298 (41.27%)	5578 (43.45%)
Fruit consumption: no	724 (5.64%)	856 (6.67%)
Vegetable consumption: yes	3574 (27.84%)	3689 (28.73%)
Vegetable consumption: no	881 (6.86%)	992 (7.72%)

*Sexual behavior*		
Active	1475 (11.59%)	1468 (11.43%)
Not active	3049 (23.75%)	3095 (24.11%)

**Table 2:** Bivariate analysis or unadjusted regression analysis for BMI status among children (12–17 years of age) in 2021.

Variable	BMI status		*P* value
	Normal weight (*n*)	Overweight/obese (*n*)	
*Adverse childhood experiences (bullying)*			<0.01
Yes	1273	709	
No	7150	3344	

*Depression*			<0.01
Yes	3244	1773	
No	5320	2341	

*Smoking cigarettes*			0.02907
Yes	181	120	
No	4604	2331	

*Marijuana*			<0.01
Yes	1276	698	
No	7266	3399	

*Fruits consumption*			0.005689
Yes	7404	3472	
No	1020	560	

*Vegetable consumption*			0.03984
Yes	4816	2447	
No	1194	679	

*Soft drink and junk consumption*			0.004848
Yes	4312	2303	
No	1898	884	

*Physical activity*			<0.01
Physically active	5245	2060	
Moderately active	2158	1240	
Physically inactive	1040	748	

*Screen time*			<0.01
Moderate screen time	5245	1240	
Active screen time	2158	2060	
No	1040	748	

*Oral health*			<0.01
Regular	6923	3046	
Occasional	328	258	
No	629	438	

Mental health (stress and anxiety)			<0.01
Yes	1708	1074	
Occasional	3175	1483	
No	1403	672	

Sleeping time			<0.01
4–6 hrs	3138	1697	
7–8 hrs	3343	1392	
>8 hrs	392	172	

*Sexual behavior*			0.04756
Sexually active	1911	1032	
Sexually inactive	4120	2024	

*Weight change status*			<0.01
Want to gain	1205	93	
Want to lose	1498	1796	
Not sure	1794	432	

*Alcohol consumption*			0.006111
Yes	732	314	
No	5448	2848	

*Sex*			<0.01
Male	4392	2264	
Female	4278	1902	

*Race*			<0.01
American Indian or Alaskan	152	118	
Native Asian	497	170	
Black/African American	1108	765	
Multiple race	1168	700	
Native Hawaiian/Hawaiian or another Pacific Islander	85	62	
White	5660	2351	

*Age*			<0.01
≤12	7	2	
13	29	8	
14	1780	772	
15	2408	1055	
16	2325	1179	
17	2121	1200	

**Table 3:** Multivariable logistic regression using stepwise selection.

Variables	OR	*p* value	95% confidence interval
*Age*			
17	Ref		
14	0.560	<0.01	0.456–0.686
15	0.788	<0.01	0.660–0.939

*Sex*			
Female	Ref		
Male	2.352	<0.01	2.035–2.718

*Race*			
White	Ref		
American Indian or Alaskan Native	1.628	0.02	1.075–2.464
Asian	0.406	<0.01	0.295–0.559
Black or African American	1.980	<0.01	1.648–2.379
Multiple race	1.203	0.04	1.001–1.445
Native Hawaiian or other Pacific Islander	1.963	0.02	1.073–3.591

*Alcohol*			
No	Ref		
Yes	0.676	<0.01	0.537–0.850

*Marijuana*			
No	Ref		
Yes	1.229	0.03	1.017–1.485

*Weight change status*			
Loss	Ref		
Gain	0.041	<0.01	0.031–054
Not sure	0.170	<0.01	0.146–0.197

*Eats vegetables*			
No	Ref		
Yes	1.196	0.03	1.013–1.412

*Physical activity*			
Not active	Ref		
Active	0.634	<0.01	0.524–0.767
Moderate	0.737	<0.01	0.603–0.900

*Oral health checkup*			
Not regular	Ref		
Occasional	0.883	0.47	0.631–1.237
Regular	0.687	<0.01	0.547–0.864

## Data Availability

The secondary dataset used for this study was collected from the CDC Youth Risk Behavior Surveillance System dataset, 2021, at https://www.cdc.gov/healthyyouth/data/yrbs/data.htm#national.

## Abbreviations

CDC: Centers for Disease Control and Prevention
YRBSS: Youth Risk Behavior Surveillance System
FV: Fruits and vegetables
OSA: Obstructive sleep apnea
GER: Gastroesophageal reflux
BMI: Body mass index
OR: Odds ratio
CI: Confidence interval
CAMK2: Calcium/calmodulin-dependent protein kinase 2
SDOH: Social determinants of health
SES: Socioeconomic status
ACEs: Adverse childhood experiences.
NIDDM: Non–insulin-dependent diabetes mellitus.
HTN: Hypertension.
FLD: Fatty liver disease/hepatic steatosis.
